# A Review of Suggested Mechanisms of MHC Odor Signaling

**DOI:** 10.3390/biology11081187

**Published:** 2022-08-07

**Authors:** Manfred Milinski

**Affiliations:** Max Planck Institute for Evolutionary Biology, 24306 Plön, Germany; milinski@evolbio.mpg.de

**Keywords:** major histocompatibility complex, signaling, olfaction, perception, microbiota, mate choice, haplotype choice, optimal MHC

## Abstract

**Simple Summary:**

Immune genes of the vertebrate MHC vary among individuals. Each individual collection is optimally diverse to provide resistance against some infectious diseases but not too diverse to cause autoimmune diseases. MHC-dependent mate choice aims for optimally complementary MHC alleles. Each potential partner signals through body odor his/her MHC alleles. Identifying the signal molecules was a long-lasting puzzle solved only recently after many deviations as described. Commensal microbiota which are controlled by the individual MHC genes differ among individuals. They were suspected repeatedly to provide the signal, though mice raised germ-free could still smell MHC genes. Carrier hypotheses came in various versions, centered around the specificity of each MHC molecule for binding peptides from diseases, shown to T lymphocytes to induce the immune response. Volatiles of various origins were suggested to fill the place of the peptide and thus reflect the identity of the MHC molecule. Finally, the bound peptides themselves were identified as the sought info-chemicals. Synthesized peptides affect mate choice as predicted. Specific olfactory neurons were shown to react to these peptides but only to the anchors that define the binding specificity. Even eggs choose sperm to produce offspring with optimal MHC, though the signaling pathway needs further research.

**Abstract:**

Although an individual’s mix of MHC immune genes determines its resistance, finding MHC-dependent mate choice occurred by accident in inbred mice. Inbred mice prefer MHC dissimilar mates, even when the choice was restricted to urine. It took decades to find the info-chemicals, which have to be as polymorphic as the MHC. Microbiota were suggested repeatedly as the origin of the odor signal though germ-free mice maintained normal preference. Different versions of the ‘carrier hypothesis’ suggested MHC molecules carry volatiles after the bound peptide is released. Theory predicted an optimal individual MHC diversity to maximize resistance. The optimally complementary mate should be and is preferred as several studies show. Thus, the odor signal needs to transmit the exact information of the sender’s MHC alleles, as do MHC ligand peptides but not microbiota. The ‘MHC peptide hypothesis’ assumes that olfactory perception of the peptide ligand provides information about the MHC protein in a key-lock fashion. Olfactory neurons react only to the anchors of synthesized MHC peptides, which reflect the binding MHC molecule’s identity. Synthesized peptides supplemented to a male’s signal affect choice in the predicted way, however, not when anchors are mutated. Also, the human brain detects smelled synthesized self-peptides as such. After mate choice, the lottery of meiosis of randomly paired oocyte and sperm haplotypes would often produce MHC non-optimal offspring. In sticklebacks, eggs select MHC-compatible sperm, thus prefer the best combination close to the population optimum.

## 1. Introduction

Immune genes of the Major Histocompatibility Complex (MHC) are by far the most polymorphic genes in vertebrates. Only one or a few different MHC alleles provide resistance to a specific parasite [1,2]. Each individual’s mix of MHC alleles determines its specific resistance against current infectious diseases [2]. This fact invites hypotheses on MHC-dependent mate choice. However, it was inconceivable until 1975 that an individual’s MHC genes organize its mate choice decisions. This year technicians at the Sloan-Kettering Institute in New York made a remarkable observation: “The technicians looking after these mice reported that the male and female of dissimilar H-2 type appeared to consort with one another to the relative exclusion of the female whose H-2 type was the same as the male’s” [3]. Starting with Yamazaki et al. [4] numerous studies, mostly with inbred strains of mice, showed that actual mate choice favors MHC dissimilar individuals e.g., [5,6,7,8,9,10,11,12,13]. MHC-dependent mate choice was demonstrated also in other vertebrates such as rats [14,15,16,17], birds [18,19,20,21,22,23,24,25,26,27], fish [28,29,30,31,32,33,34,35,36,37], reptiles [38] small mammals [39], primates [40], and humans [41,42,43,44,45,46].

This review follows the history of studying MHC-dependent mate choice. Because most of the early experiments were performed with mice, later followed by stickleback fish, insights and ideas focused to some extent on these models and thus had limitations for being generalized. It is, however, convenient to describe the history of understanding mechanisms and functional consequences of MHC-dependent mate choice in one or two finally well-studied and understood systems rather than including the great variation among species right from the beginning. I found it convincing how the various parts of a system made sense as predicted by immunological theory. It could be the ‘null system’ which needs to be extended to cover natural variation.

One sensory modality that transports the information about an individual’s MHC was revealed again in mice: odor. The choice between urine odor from MHC similar and dissimilar mice in a y-maze favored MHC dissimilar individuals e.g., [5,47,48,49,50,51,52,53,54]. Thus, odor transmits the MHC signal and the choosing mouse knows her own MHC otherwise she could not prefer ‘dissimilar’. At the time some MHC researchers were skeptical. ‘Some immunologists resist the idea that MHC genes could themselves specify odors. In part, this is because there has been suggested no plausible mechanism by which these genes, which code for cell-surface proteins, could also specify differential body odor’ [55,56]. A number of studies revealed mate choice that was based on odor, in rats [14,15], fish [29,30,31,32,33,34,36], reptiles [38], birds [24,25,26], small mammals [57], and Humans [42,43,44,45,46,58]. Thus, there must be a mechanism. Because the polymorphic MHC genes of the vertebrate immune system are highly conserved and at least 450 my old [59], the olfactory signaling and recognition system may probably be similar in all jawed vertebrates.

## 2. The Microbiota Signalling Hypothesis

The puzzle of the origin of the odor and of its composition took a long time to be solved. One possibility could be that populations of commensal microorganisms generate differential odorants whose composition eventually reflects MHC diversity [49]. The MHC class II gene family may be a candidate for adjusting diverse and host-specific microbiota. MHC genes may affect the composition of the microbial community of symbiotic bacteria through the elimination of specific bacterial species in an antigen-mediated fashion; thus, an individual’s MHC genotype might be able to shape the composition of symbiotic bacteria that can survive on or inside the host. The microbial communities that result potentially influence host odor [24,26] as well as host fitness [60].

Bolnick et al. [61] showed that MHC IIb polymorphism is correlated with variation in gut microbiota among individuals within a single population of three-spined sticklebacks. Individuals that had more divergent MHC motifs carried less diverse microbiota. However, MHC explained roughly only 10% of microbial variation [61]. The primary source of body odor in birds is preen oil [25,26]. Similar preen secretion chemicals correlate positively with MHC-relatedness [25]. In behavioral discrimination tests, kittiwakes and blue petrels can assess MHC similarity on the basis of odor [25]. When song sparrows (Melospiza melodia) were presented with preen oil from conspecifics of opposite-sex, both sexes preferred odor from MHC- dissimilar to MHC-similar birds in a two-choice design [26]. According to the authors, song sparrows can discriminate MHC similarity of potential mates by using preen oil odor. Similarity at MHC is thus a predictor of similarity in the composition of preen oil [62]. This relationship may be hypothesized to be mediated by symbiotic microbes. The MHC genotype, the microbial communities in preen glands, and the chemistry of preen oil was characterized in song sparrows [62]. Pairwise MHC similarity predicted similarity of microbiota in preen glands. However, the overall similarity of microbes did not predict similarity in the chemistry of preen oil.

Obviously preen oil contains an MHC-dependent odor signal. Because preen oil composition was related more strongly to MHC genotype than to preen gland microbiota overall, the authors suggested the following: the effects of MHC on the composition of preen oil are not facilitated primarily through microbiota in the preen gland [62]. Instead, the MHC genotype may affect host odor more directly [62]. Although microbiota within preen glands correlate with MHC, which might be just MHC’s immunological action of regulating bacteria, it is not clear whether microbiota contribute to MHC dependent odor of preen oil.

The microbiota hypothesis experienced almost continuous ups and downs that read similar to a fascinating criminal story: Yamazaki et al. [49] suggested that populations of commensal microorganisms may generate differential odorants; their composition maybe somehow adjusted to MHC diversity. In the same publication, they provide a direct test of the hypothesis by excluding microbiota. If microbiota are necessary for producing the MHC-dependent odor signal, germ-free mice should not produce it. This hypothesis was tested: Mice are trained in a Y-maze system to distinguish the urinary odors from MHC-congenic mice. Mice could also easily be trained to distinguish the urines of MHC-congenic mice that had been raised germ-free. Also, mice that had learned to distinguish the urines of conventionally maintained MHC-congenic mice were shown to distinguish readily the urines of germ-free congenic mice. Thus, MHC-determined odor types do not depend on microorganisms generating odorants [49]. In the same year, Singh et al. [63] published the finding that rats lost their individuality odor when reared in a germ-free environment. Male rats were reared in a germ-free environment after being born by the cesarian section. A habituation-dishabituation test revealed that urine from the germfree rats was not discriminated, whereas urine from rats of the same strain that were housed conventionally could be discriminated. When urine from germfree rats was collected, after they had been moved to a conventional animal house after recolonization with commensal flora, it was discriminated against. This indicated that bacteria had an essential role in determining the urinary odors of MHC congenic rats. ‘We would predict that bacteria may control the production of MHC-specific odors in mice and other species as they do in rats’ [17].

The results of Yamazaki et al. [49] and Singh et al. [17] are at variance. Singh [63] offers a solution for the discrepancy. “We suspect that the differences between the results obtained lies in the methodology used, ’habituation-dishabituation’ relies on the odor stimulus being different enough for it to be interesting to the responder animal, leading to its dishabituation, while the motivation to detect a difference using the ‘training-reward’ system is much greater: the mice are thirsty and detecting the difference allows them to drink. Thus, the residual MHC-related odors (derived from normal metabolic processes, other than gut flora) that remain in germ-free urine are more likely to be detected by the trained mice, while untrained mice may not have the ‘motivation’ to discriminate between the germ-free urine samples.”

Yamazaki et al. [56] comment on ‘this apparent contradiction’ that the clear key are differences in methods. They assume that the relevant odorants are still expressed in germ-free animals but in smaller amounts such that they fail in the habituation paradigm to motivate non-contingent investigation. ‘If this is so, then the hypothesis that MHC genes specify odor by controlling commensal microflora cannot be true’ [56].

If the ‘microbiota signaling hypothesis’ is to be revived, some proximate and functional questions need to be answered, for example [64]: If we consider an info-chemical X that animal-A produces and which alters the behavior of animal-B, to demonstrate that the microbiota in animal-A synthesize info-chemical X, three lines of evidence are required: (i) Some microbiota in animal-A can synthesize X. (ii) If the microbiota from animal A are eliminated, both the loss of X and the loss of the behavioral trait of animal-B results; and (iii) interaction with microbe-free animal-A that has been supplemented with the info-chemical X revives the behavior of animal-B.

If these stringent conditions are fulfilled, it needs the study of the evolutionary processes that facilitate the origin and also the persistence of communication that is microbial-mediated, rather than chemical signals that the animal host synthesizes itself, avoiding conflict between microorganisms and the animal host [63,64]. Before we come back to microbiota we discuss what was proposed next.

## 3. The ‘Carrier Hypothesis’

Several ideas assumed that MHC molecules are the carriers of the specific info chemicals. After an infection, the host’s cells contain foreign proteins which are degraded by proteasomes into small pieces, about nine amino acids long, called peptides. In order to inform the T lymphocytes outside the cell, the peptides need to be bound and transported through the cell membrane and presented to T lymphocytes. This is the task of MHC molecules if the peptide fits into the binding groove of an MHC molecule by its ‘anker amino acids’ in specific positions of the peptide [65], irrespective of whether it is a foreign or a self-peptide. Each of an individual’s few MHC alleles has its specific binding groove to bind only specific peptides, although examples of promiscuous peptide binding exist [66,67]. To avoid auto-immune diseases, T lymphocytes have been selected in the thymus to recognize only foreign peptides to induce an immune response [68]. Thus, the broader the spectrum of one’s MHC alleles, the more infectious diseases can be presented to and attacked by the immune system suggesting mate choice for MHC dissimilar partners. This allows each MHC molecule to bind and carry only chemicals that transport information of the MHC molecule’s individuality expressed by its peptide binding groove. From collecting all the peptides transported by the MHC molecules of an individual it would be possible to deduce the nature of that individual’s MHC alleles, i.e., its MHC genotype. Thus, peptides would be ideal info-chemicals constrained by their low volatility.

However, other info chemicals carried by MHC molecules were suggested. Evidence for volatiles that have distinctive patterns according to MHC type has been reported [54]. Carboxylic acids have been found, in behavioral active dimethyl ether extract of acidified urine, that distinguish male mice that differ only at their MHC [55]; these chemicals probably have a critical role when MHC-congenic mice are discriminated by olfaction [55]. It is suggested that the most likely mechanism for this could be that circulating odorants are bound selectively by soluble MHC gene products themselves; these have presumably lost their bound peptide before. Then the odorants are released to a minimal degree in serum and probably more extensively during renal processing and excretion. It is suggested that these odorants are likely the volatile acids that have been identified or precursors of them [56].

The fact that MHC class I molecules can associate selectively with small molecules could also suggest a way for transporting a unique mixture consisting of volatile, endogenous metabolites to urine from the blood by MHC glycoproteins [16]. Although each individual has a similar metabolic pool, this mixture would be unique to the transporting particular MHC molecule. An individual-specific odor would be imparted to the urine. The postulated volatile molecules need to be identified [16]. Maintaining the microbiota hypothesis, Singh et al. [17] propose that the excretion of class I molecules has an important role in individual odor to be determined in the urine. Bacteria are considered to be an essential source of the body’s pool of odorant molecules by which individuals do not vary. A mixture of odorants is selected by an MHC molecule from the body pool. The MHC molecule acts as a carrier to deliver the cocktail to the urine. This is assumed to be analogous to the manor of MHC molecules binding and presenting immunogenetic peptides to T lymphocytes [17]. ‘The ‘carrier hypothesis’ represents the simplest explanation of the mechanism whereby the MHC confers odors of genetic individuality’ [64].

However, Singh [69,70] dismisses the microbiota hypothesis: The intimate linkage of MHC genotype with the urinary odor was argued to be indirect and to reflect the immune response that responds to commensal bacterial flora causing individual MHC types to be associated with unique flora. The volatile odorants excreted were assumed to be secondary metabolites that are derived from these organisms. This hypothesis seemed unconvincing a priory because the types and relative numbers of commensal bacteria are required not to vary over time, which is not true [71]. According to three studies, immune regulation of commensal flora is not needed for the determination of MHC-associated odors [49,72,73].

Singh [69,70] again supports the carrier hypothesis in more detail. He argues that MHC molecules are not likely to be the odor components because of their size and missing vapor pressure. However, MHC molecules might be in an allele-specific association with smaller molecules for transporting them to the urine [16,69]. Thus, from a pool of metabolites, a unique mixture of volatiles could be selected, in which, commensal flora could take part [17]. After transport, a unique odor that is MHC specific would appear in the urine. A mechanism might work by which MHC molecules each pick up a unique mixture of volatiles in their binding groove to be transported to the urine as an individuality marker.

MHC-associated peptides are cleared into the circulation, undergoing further fragmentation. The release of any bound peptides allows the now empty platform to bind a unique mixture of odorants, to be transported to the urine where they are further degraded to molecules that make up an odor that is MHC specific. “The nature of the specific odorant molecules that are bound to soluble class I molecules is unknown, although carboxylic acids may be one source” [54].

“The problem with the carrier hypothesis is that it is difficult to imagine how binding properties of MHC molecules might be converted from being hydrophilic peptides-binding molecules to hydrophobic aromatic-binding molecules” [52]. Even if one would agree with the “empty platform” being able to bind a cocktail of volatiles, how could the released molecules of the ‘cocktail’ transport the information content of the specific sequence of amino acids mirroring the binding cleft of the MHC molecule? A receptor in the receiver animal needs to catch and reassemble the loose volatile molecules into the original sequence to extract the individuality of the source MHC molecule. It needs the study of the mechanisms of the receptor side. We are not yet informed about the nature of the involved volatile molecules. No qualitative differences in volatile compounds have been found in association with MHC types by any investigation except one. However, for volatile metabolites, patterns or relative ratios vary in relation to MHC types, but in a way that is inconsistent and complex [74].

## 4. Mate Choice Optimizing MHC for the Offspring

The first results of mate choice experiments, e.g., Ref. [4] found a preference for MHC dissimilar mates, which would generate maximally heterozygous offspring being potentially resistant against many infectious diseases. Because having several MHC loci is better than having one, there should be far more MHC loci than the existing ones. Duplication of loci could easily be accomplished [75]. Sex would not be necessary for maximizing resistance if everybody has the full spectrum of MHC variants that exist in the vicinity. However, with each new MHC molecule added to the repertoire of an individual, each T cell line that detects self-peptides bound by that molecule must be eliminated to avoid auto-immune diseases. The low number of MHC loci of, e.g., humans and mice, could to be optimal for balancing an increased number of foreign peptides to be presented and an increased number of T cells eliminated from the original repertoire [68,76].

Theory based on immunological results predicted an optimal number of different MHC molecules per individual instead of maximizing MHC diversity [77,78]: with an increasing number of different MHC alleles per individual, auto-immune responses become more likely against cells with MHC molecules presenting self-peptides [79]. For avoiding auto-immunity such T cell clones are removed (negative selection) already in the thymus. Consequently, the elimination of T cell clones reduces the body’s ability to fight infections. Autoimmune responses do not appear, if negative selection works properly. Finally, with too many MHC molecules, no T cells exist exposing the individual unprotected to its diseases. The inevitable consequence is an intermediate (optimal) number of different MHC alleles per individual, enough to fight many natural diseases and not too many to retain a ‘reasonable’ collection of T cell clones. The optimal number should and does vary among species dependent, e.g., on the range of natural diseases [80]. With only one existing disease, although an unrealistic assumption, the optimum would be one MHC molecule per individuum. Furthermore, the optimal number of MHC molecules should include the one that provides resistance against a disease that has just become common. Females can find the respective male by performing a ‘health test’ of its vigor. Males with ‘revealing handicaps’, e.g., costly conspicuous colors, loud songs, etc. are healthy and should be and are preferred by females [81,82]. All this has been shown operating in sticklebacks. Numerous other examples exist. This is part of the ‘null model’, which predicts complementary MHC-dependent mate choice with variations of this basic scheme. We found in sticklebacks a higher MHC polymorphism in lakes than in nearby rivers combined with a higher range of natural parasites in lakes than in rivers corresponding with a lower optimum in rivers than in lakes [80]. Surprisingly females chose mates such that their offspring would have the local individual optimum number of different MHC alleles [32,33]. The females have the information about their local optimum in their genes, even though the river and lake populations are interconnected—an example of sympatric speciation. I wonder what climate change will do to these local adaptions. Would additive genetic variation allow populations to track the new optima?

A reviewer suggested that a problem might arise in populations with large population variability (in terms of loci number as well as numbers of alleles in the gene pool). It can be predicted that cases where multiple alleles binding the same peptide in the sender will match a single allele in the receiver will be common, especially with promiscuous peptide binding to structurally heterogynous peptides. Whether evolution has solved this problem can be studied by detecting such cases with their fitness consequences. It could be hypothesized that populations with extraordinary high numbers of MHC loci [83,84] have high numbers of infectious diseases per individual and are additionally handicapped, though potentially at the optimum, because of many deselected T cell clones and thus lowered resistance. Are they evolutionarily on a ‘dead end road’?

**Figure 1:**
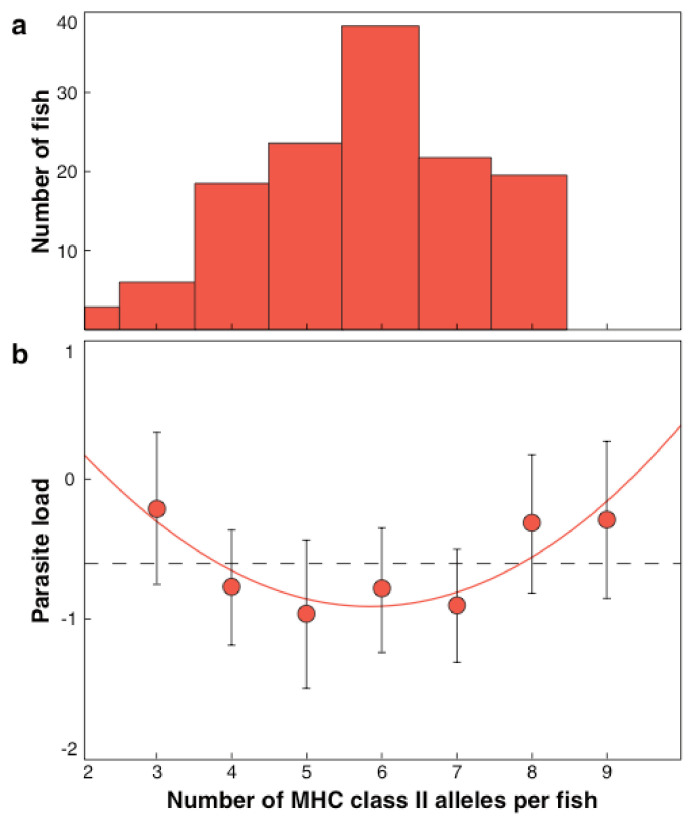
(**a**) Frequency distribution of the number of MHC class II alleles detectable in 144 three-spined sticklebacks from a lake system. The mean number of MHC alleles was 5.8 (from [29] Reusch et al. 2001). (**b**) Relationship between the number of expressed MHC class II molecules and mean parasite load, expressed as summed residuals from general linear model (GLM) analysis. The function matches a quadratic polynomial with a minimum of 5.82 alleles. (from [86] Wegner et al., 2003a).

An optimal individual MHC diversity is predicted [77,78,79]. The first immunogenetic optimum found in nature was published on three-spined sticklebacks [29,85] and proven by an experiment (Figure 1) [86]. This optimal MHC per individual maximizes reproductive success during a lifetime [87] and increases survival under natural conditions [88,89]. Also, for other vertebrates, e.g., birds [21,22,89], trout [37], and voles [39] optimal individual MHC diversity was demonstrated.

Experimental mate choice studies found that female three-spined sticklebacks prefer the odor of the male that signals the possession of MHC alleles together with the female’s approach to the individual optimum as predicted [29,30,31,90].

## 5. Inbred Mice and Hutteries Confirm the Rule in Different Ways

Why did the studies with inbred mice usually find a preference for MHC dissimilar mates, contrary to the optimality prediction? When inbred female mice had the choice between two inbred males, one being slightly more heterozygous, she can approach the optimum for her offspring only by preferring the dissimilar male, because inbreeding produces homozygous mice below the optimum. This is reminiscent of a population with low MHC allele diversity, e.g., after a population bottleneck. When this scenario was staged experimentally using wild-caught females that had only a few MHC class-IIB alleles. These females were offered the choice between water taken from tanks housing a dissimilar and a male MHC genotype that was identical to the female’s MHC genotype, both had the identical low number of MHC alleles as had the female, and the females behaved indeed similar to inbred mice and preferred the dissimilar male [90]. Sticklebacks originated from the same but outbred population where optimizing had been found [29]. In both studies, females need to ’self-refer’ to their own MHC alleles to ‘calculate’ the combination they would achieve with the male on offer that needs to be most complementary to approach the optimum.

In Hutteries, a North American reproductive isolate of European ancestry, fewer matches of HLA haplotypes were found between spouses than expected, implying that Hutterie mate choice within their population is influenced by HLA haplotypes, with avoidance of spouses with haplotypes that are the same as one’s own, i.e., a preference for ‘different’ [42]. In humans, the MHC is called HLA (Human Leucocyte Antigen). In a further study [45] forty-nine Hutterie women were asked to evaluate the odor of t-shirts worn by six men of diverse ethnicity that differed from that of the Hutterie community, but who nonetheless carried some HLA alleles found in the community as well as completely foreign alleles (median of two allele matches between smellers and the odor donors). The women were asked which odor they would choose. There were no absolute preferred odors, but significant differences relative to each woman. The donor of a woman’s most preferred odor, rated as more pleasant, had significantly more HLA allele matches with her own alleles than did the donor of her least preferred odor. Interestingly, a woman’s choices were based on matches to the alleles inherited from her father rather than from her mother. These results appear to be at variance with previous findings of human HLA-dependent mate choice where donors with different alleles were preferred [42,43]. Neither appears to support optimizing individual HLA (MHC). However, the earlier studies [42,43] study involved choice within a closed community, both potentially below the optimal HLA (MHC) individual diversity. Jacob et al. [45] investigated choices among communities, within the lower range of allele matches; the results show that women avoid odors from donors with zero or one HLA allele matches to their own HLA alleles and prefer odors from donors with more HLA matches. Donors from different ethnicities might allow an approximation of the optimal diversity ‘from below’ for the offspring when they carry at least a few matching alleles, which may be an optimal evolutionary strategy to preserve the immunocompetence of offspring [45].

## 6. The ‘MHC Peptide Hypothesis’

An odor signal is needed that transmits the exact information of the sender’s MHC alleles. MHC ligand peptides fulfil this requirement [91,92]. MHC molecules are specialized carriers of peptides and devices to display them. In the cell peptides that are derived from processes that degrade foreign and self-proteins are fixed on MHC molecules. These complexes are moved to the surface of the cell. The composition of the peptide mirrors the binding specificity of the MHC molecule. The ‘MHC peptide hypothesis’ assumes that smelling the peptide ligand (‘the key’) informs about the MHC protein (‘the lock’) [93]. From its peptide ligands, an individual’s underlying MHC diversity can be derived. Peptide MHC complexes move from the cell surface to the extracellular space, where the peptides are released in bodily fluids. They could interact with receptors of the olfactory system [94]. Peptides activate olfactory sensory neurons even at low concentrations [95,96]. They could be demonstrated to be present in urine [97]. Since the mix of peptides carried by the MHC molecules of an individual reflects the mix of the individual’s MHC alleles, the peptides themselves, transported by its MHC molecules could be the polymorphic signal. ‘The role of MHC peptides as signals of individuality appears to be evolutionarily conserved’ [91].

## 7. Experimental Tests of the ‘MHC Peptide Hypothesis’

**Figure 2:**
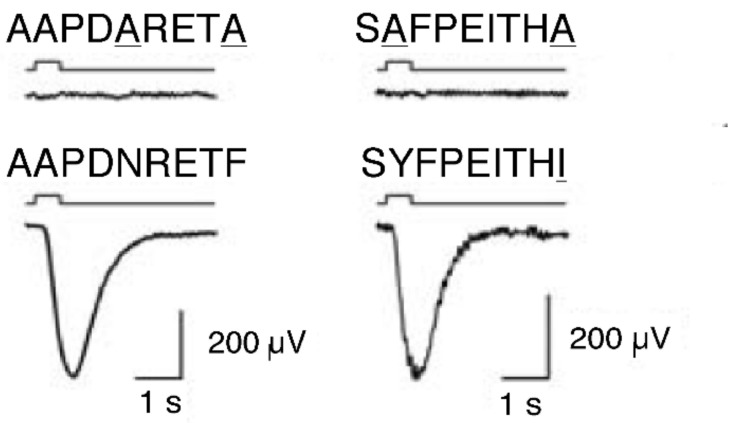
Electrical response of vomeronasal neuro-epithelium to synthesized MHC ligand peptides (lower graph): e.g., AAPDNRETF is the sequence of amino acids of the peptide, for example, A stands for Alanine. Line below indicates the start of the stimulus. Below a complete receptor potential occurs. Electrical response of vomeronasal neuro-epithelium to peptides with anchors (underlined) mutated to alanine, which never serves as an anchor (upper graph). No receptor potential occurs. (from [94] Leinders-Zufall et al., 2004).

MHC ligand peptides have the function to stimulate some specific olfactory neurons in the mouse vomeronasal organ [83], and also in the mammalian main olfactory system [96]. Each individual neuron responds to one specific MHC ligand peptide only. This specificity is defined by the peptide’s anchor residues that allow binding to ‘its’ MHC molecule. Neurons do not distinguish between peptides that have identical anchors but differ otherwise. However, the neuron did not respond to mutated peptides with only their anchors mutated to alanine; alanine is never an anchor (Figure 2). The olfactory neurons take exactly those parts of the peptide into account that define the MHC molecule’s specificity of binding the peptide.

Are MHC peptides used for social recognition? Bruce [97] detected pregnancy failure when a female mouse that was recently mated and the stud male removed, was presented with a new male that originated from a different strain (or when she was provided with the new male’s odor). Yamazaki et al. [98] found female pregnancy block when the odors came from two congenic males that differed by only one MHC class I allele. The “Bruce-effect” could be induced also when the mated female was presented with only one MHC peptide that was specific to the other strain. She did not block pregnancy when presented with an MHC peptide specific for her own strain [94]. MHC ligand peptides thus function as signals of individuality in mice [92].

## 8. MHC Ligand Peptides Are Used in Actual Mate Choice Decisions

**Figure 3:**
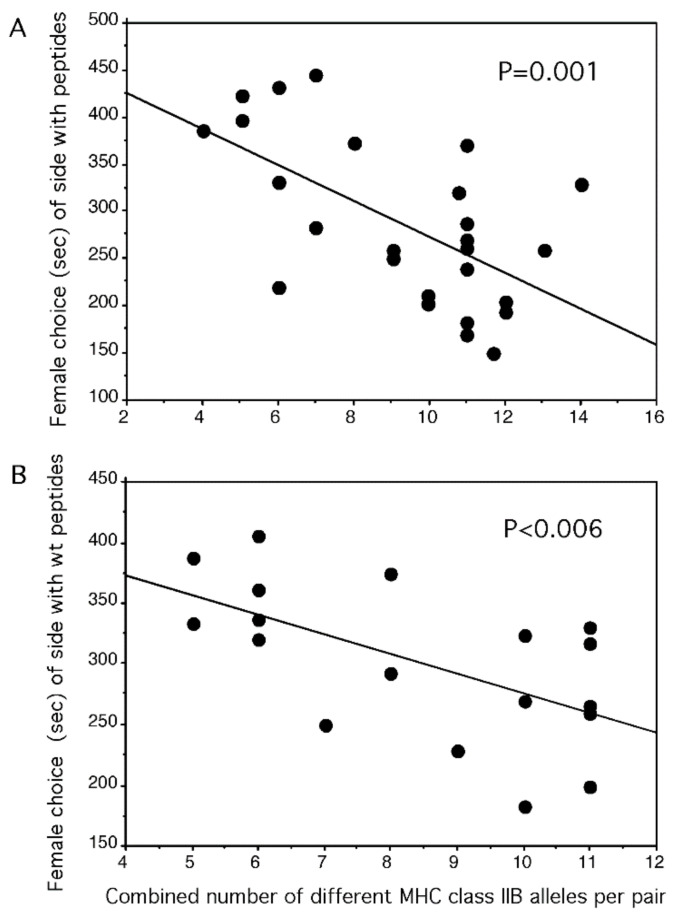
In a two-choice flow channel, single gravid female three-spined sticklebacks chose between (**A**) the water from the tank of a single male supplemented with solvent only or four peptides in a solvent. The time (a total of 600 s) females spent in the side of the flow channel to which peptides were supplemented is shown as a function of the combined number of different MHC class II alleles of the pair. The offspring of a pair with a combined diversity of 10 alleles would have about five alleles because each parent has haploid gametes. (**B**) The water from the tank of a single male supplemented with four peptides in solvent or four mutated peptides in a solvent. The anchors of mutated peptides were exchanged with alanine, which never serves as an anchor. The time (a total of 600 s) females spent in the side of the flow channel to which wildtype peptides were supplemented is shown as a function of the combined number of different MHC class II alleles of the pair. (from [30] Milinski et al., 2005).

If peptides naturally signal via odor revealing the MHC alleles of a male, it should be possible to manipulate the information of the signal by adding further peptides. Because a female stickleback prefers a male that would offer by scent MHC alleles that optimally complement her own alleles [90,99], a suboptimal male’s attractiveness should be increased and an optimal, as well as a super-optimal male’s attractiveness, should be decreased by adding the same four synthetic MHC peptides to either male’s natural signal. When choosing between spiked and un-spiked water from the tank of the same male, the peptide side was preferred when the pair had a combined diversity that was below the optimum, but the spiked side was avoided when the pair was above or at the optimum [30] (Figure 3). However, the same synthetic peptides were ignored when their anchors were mutated to alanine and they were offered against the unmutated wildtype peptides on the other side of the flow channel [30]. The females reacted only to the wildtype peptides and treated the mutated peptides the same as a solvent only as in Figure 3B.

As do mouse olfactory neurons, sticklebacks take only the peptides’ anchors into account. Both in sticklebacks and mice MHC ligand peptides interact as predicted with natural signals that inform about MHC. MHC peptides may be part of the natural perfume-like signals in other vertebrates including humans.

## 9. MHC Peptide Ligands as Olfactory Cues in Humans

Psychometric tests showed [58] participants preferred their modified body odor, when their own synthesized ‘self’ peptides were offered, to modification by synthesized ‘nonself’ peptides (from another participant), when asked their decision whether what they smelled was perceived as ‘like themselves’ or ‘like their favorite perfume’. Thus, MHC peptide ligands may function as part of body odor in humans. These findings are reminiscent of previous results showing that humans that share specific MHC alleles have also a similar preference for the same natural ingredients of perfume [100]. Perfumes may contain diverse peptide mimics. A ‘functional magnetic resonance imaging’ study found that self-peptides that were allele-specific activated a region in the right middle frontal cortex, demonstrating the human sensory facility to recognize odor cues that are specific to MHC [58]. Therefore, peptides possibly invoke sensory neurons in the main olfactory epithelium, as was the case in mice [96]. Activation of the brain through exposure to peptides does not mirror the precise chemical structure of the peptides but rather their qualities of ‘self’ or ‘nonself’ in relation to the MHC genotype of the individual. Probably there is an internal reference for the genotype of MHC, reminding us of a similar finding in mice [94] and sticklebacks [30]. As in mice and fish, the sensory evaluation of the diversity of MHC through recognizing MHC ligand peptides that are structurally diverse may be part of human MHC-dependent behavior.

## 10. Can Signaling Microorganisms Help Optimizing?

In wild sticklebacks, individuals that possess more divergent MHC motifs harbored less diverse microbiota, although MHC explained roughly only 10% of microbial variation [61]. An estimated optimum of microbial variation would therefore be extremely vague, in addition, microbial variation fluctuates with diet and many other environmental influences. By preferring a mate with more diverse microbiota, a female would select the more MHC dissimilar mate. Thus, for choosing ‘dissimilar’ using signals from microbiota would suffice, in principle. However, for selecting the optimally MHC complementary mate, it needs ‘smelling’ exactly which MHC alleles a potential mate offers. A male would need to harbor exactly only those microorganisms that signal his array of MHC alleles. He carries, however, vastly more different microorganisms than this small number. Thus, for optimizing MHC using odor from microorganisms cannot work.

## 11. A Failed Revival of the Microbiota Signaling Hypothesis

Schubert et al. [101] undertook the impressive work of reviewing 577 publications to find how the MHC might mediate social odor via the microbiota community, for example, with regard to mate choice that is MHC-dependent. None of the 577 studies found, however, the odor being a social signal. Their extensive review of the complex immunological network potentially affecting microbiota and odor through various pathways did not solve the problem of how the microbiota of an individual could signal the possession of exactly its MHC alleles allowing for optimal mate choice. Nevertheless, the authors “hoped that their review stimulates advances in the investigation and understanding of this key pathway for social communication” [101]. A response [102] to a commentary [103] pointing to gaps in their argumentation omitted responding to the main criticisms [102]. On the contrary, the authors reiterate that although “an established mechanism that provides allele-specificity has already been identified: peptide ligand-based odor signals” [101], multiple signaling mechanisms that transmit the same information are useful [101]. Two counter-arguments can be listed: (i) Microorganisms cannot transmit the same information (see above), and (ii), if a microbiota signaling mechanism would join highly conserved peptide signaling [91], if microbiota could do the signaling, this additional second mechanism cannot evolve because it does not increase the sender’s performance compared with the primary mechanism–no fitness gain, no evolution. No need for keeping alternative hypotheses on the table.

## 12. Egg Chooses Sperm with Regard to MHC

Precopulatory selection for mates with complementary whole MHC genotypes allows only poorly approaching the best match. A maternal and a paternal haplotype are randomly combined, which results from meiotic segregation among both oocytes and sperm, phenotypically unpredictable. This determines ultimately the genotype of the offspring. The lottery of random fusion of gametes can easily produce nonoptimal combinations of MHC haplotype. If the egg chooses and prefers the more complementary of the two sperm haplotypes of a male, it has an offspring that is closer to the optimum MHC diversity. The expectation that sperm may select for MHC complementarity has been hypothesized [104,105,106,107,108]. Some studies found MHC expression on the sperm surface [2,109].

How does the egg decide? Sperm–egg interaction leads to cross-talks, studied in mice which clearly showed expression of MHC class II antigen on the postacrosomal membrane of the sperm head in mice. The roles of the MHC class II molecule of sperm in fertilization were studied by an in vitro fertilization (IVF) system. In the late stage of fertilization (i.e., fusion stage of sperm postacrosomal membrane and egg plasma membrane), the immuno-related molecules participated in fertilization [110,111]. Haploid expression of MHC class II molecule on the sperm head was demonstrated [112]. This molecule has an adhesive role during the adhesion and fusion of sperm and egg in the posterior region of sperm. The expression of CD4 molecules on the plasma membrane of murine eggs was demonstrated which corresponds to MHC class II molecules on the head of sperm [111]. Further research is needed.

The micropyle of fish eggs, which is an opening in the coat of the egg through which sperm enter the egg for fertilization, apparently to carries specific molecules [113]. These play a role in attracting sperm toward the opening of the micropyle. Its removal reduces fertilization success significantly [114,115]. This mechanism was species-specific as tests showed, suggesting that the molecular mechanism involved has a certain specificity. These observations highlight a potential way to preferentially guide MHC-complementary sperm to the micropyle opening. Though suggestive, there is still a gap to be filled.

## 13. Functional Tests Implying Choice of Gametes

When eggs could choose between sperm derived from MHC identical and from MHC dissimilar males, sperm derived from MHC identical males had greater success in fish [115,116,117]. However, in red jungle fowl, sperm derived from MHC dissimilar males was more successful in fertilizing eggs [118]. When congenic laboratory strains of mice were crossed, parental MHC haplotypes that combined nonrandomly suggested blastocysts that result. However, the process was affected by the infection of the parents with mouse hepatitis virus [119,120]. ‘Decisive experimental evidence for oocyte selection of specific sperm haplotypes is still elusive’ [121].

## 14. Oocyte Selection for Sperm Haplotype to Optimize MHC of the Zygote

**Figure 4:**
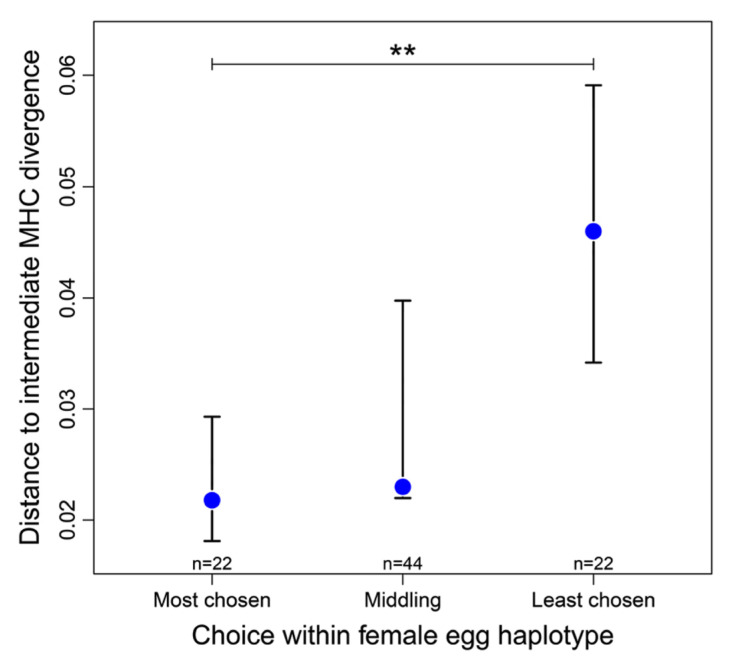
The deviation (median absolute distance ± 95% CI) between a sperm’s egg-to-sperm MHC divergence and a hypothetical population optimum (0.2356) is shown here for the most successful (‘most chosen’), the least successful (‘least chosen’) and the two middling of the four sperm haplotypes available for fertilizing a given egg haplotype. ** = *p* < 0.01 (from [122] Lenz et al., 2018).

In vitro exposure of female stickleback eggs simultaneously to equal volumes of sperm from two males, resulted in four male MHC haplotypes that were available for fertilization of each egg-haplotype, mimicking the situation with a sneaking neighbor [122]. When the four sperm MHC haplotypes that were available to a given female egg were ranked according to their success in fertilization, the most successful of the sperm haplotypes was closer than the least successful one to the mean MHC divergence of the population, which approximates the optimal individual MHC divergence (Figure 4). To avoid interaction with sperm traits, e.g., velocity or density, of the two males, only the two sperm haplotypes within each male were compared. Here sperm traits should be the same. Again, the zygote produced with the more successful sperm haplotype of two was closer to the MHC optimum than the zygote formed with the less successful one [122]. Thus, eggs selecting MHC-compatible sperm prefer the best combination circumventing the lottery of meiosis, at least in sticklebacks. The mechanics of choice may be as described [113,114]. The signal mode is not yet known, it could be odor again.

## 15. Conclusions

MHC-dependent mate choice was not known until 1975, when it was detected by chance in inbred mice. The further history of searching for a mechanism for signaling one’s MHC alleles to a potential partner revealed that it is odor in all species studied. The nature of the signal molecule, however, puzzled researchers for a long time. Microbiota were discussed and rejected repeatedly. Because they are controlled by the MHC, a negative correlation between MHC and microbiota diversity is appealing though inevitable even without a signaling function. A carrier hypothesis appeared in various versions centered on the binding specificity of the MHC molecule that was assumed to pick up and carry various substances after releasing its bound peptide. These substances should reflect the binding specificity of each MHC molecule, reassembled to signal an individual’s MHC diversity. The volatile metabolites that were suggested vary in relation to MHC types, but in a way that is inconsistent and complex.

The ‘MHC peptide hypothesis’ is a pure form of the carrier hypothesis. It assumes that the bound peptide reflects, similar to a key the specificity of the lock, and the specificity of the binding groove of the MHC molecule. Synthesized MHC ligand peptides added to a male’s signal affect the females’ choice decisions in the predicted way. MHC ligand peptides stimulate also olfactory neurons of the mouse vomeronasal organ, as well as in the mammalian main olfactory system. Mutating the anchor residues of the synthetic peptides rendered them ineffective in influencing mate choice and in eliciting a response of olfactory neurons. Since the peptides that are bound by the MHC molecules of an individual reflect its diversity of MHC alleles, the peptides emitted by the individual could be the polymorphic signal. Because vertebrate mate choice aims at an optimally complementary mate that signals exactly its MHC alleles, too many potentially signaling microbiota species per individual cannot do the job. The microbiota hypothesis, though attractive, is not supported for various reasons. Signaling individuality by MHC ligand peptides is apparently conserved evolutionarily and thus could be the MHC signal in all jawed vertebrates. The lottery of meiosis of randomly paired oocyte and sperm haplotypes would often produce MHC non-optimal offspring. As shown in sticklebacks, eggs select MHC-compatible sperm leading to a combination close to the population optimum.

A critical final conclusion: MHC-dependent mate choice may fail to be detected (i) when the assumption of preference for partners with different MHC alleles is tested although an optimal individual diversity exists in the population, and (ii) when MHC-dependent mate choice does not exist in the studied population. (i) and (ii) are undistinguishable without further study which might not have been conducted. Therefore, this review is biased because many non-fitting results might not have been published and because of my own bias of presenting a readable review by concentrating on the majority of the well-understood studies. The whole story is certainly more complex as future studies will show.

## Data Availability

Not applicable.

## References

[1] Hill A.V.S., Allsopp C., Kwiatkowski D.P., Anstey N.M., Twumasi P., Rowe P.A., Bennett S., Brewster D., Mcmichael A.J., Greenwood B. (1991). Common West African HLA antigens are associated with protection from severe malaria. Nature.

[2] Milinski M. (2006). The major histocompatibility complex, sexual selection, and mate choice. Ann. Rev. Ecol. Syst..

[3] Boyse E.A., Beauchamp G.K., Yamazaki K. (1987). The genetics of body scent. Trends Genet..

[4] Yamazaki K., Boyse E., Mike V., Thaler H., Mathieson B., Abbott J., Boyse J., Zayas Z.A., Thomas L. (1976). Control of mating preferences in mice by genes in major histocompatibility complex. J. Exp. Med..

[5] Yamazaki K., Yamaguchi M., Andrews P.W., Peake B., Boyse E.A. (1978). Mating preferences of F2 segregants of crosses between MHC-congenic mouse strains. Immunogenetics.

[6] Yamazaki K., Beauchamp G.K., Kupniewski D., Bard J., Thomas L., Boyse E.A. (1988). Familial imprinting determines H-2 selective mating preferences. Science.

[7] Yamaguchi M., Yamazaki K., Boyse E.A. (1978). Mating preference tests with the recombinant congenic strain BALB HTG*. Immunogenetics.

[8] Beauchamp G.K., Yamazaki K., Bard J., Boyse E.A. (1988). Pre-weaning experience in the control of mating preferences by genes in the major histocompatibility complex of the mouse. Behav. Genet..

[9] Egid K., Brown J.L. (1989). The major histocompatibility complex and female mating preferences in mice. Anim. Behav..

[10] Eklund A.E.K., Brown J.L. (1991). The major histocompatibility complex and mating preferences of male mice. Anim. Behav..

[11] Eklund A.E.K. (1997). The major histocompatibility complex and mating preferences in wild house mice. Behav. Ecol..

[12] Potts W.K., Manning C.J., Wakeland E.K. (1991). Mating patterns in seminatural populations of mice influenced by MHC genotype. Nature.

[13] Potts W.K., Manning C.J., Wakeland E.K. (1994). The role of infectious disease, inbreeding and mating preferences in maintaining MHC diversity: An experimental test. Phil. Trans. R. Soc. Lond..

[14] Brown R.E., Singh P.B., Roser B. (1987). The major histocompatibility complex and the chemosensory recognition of individuality in rats. Physiol. Behav..

[15] Brown R.E., Roser B., Singh P.B. (1989). Class I and class II regions of the major histocompatibility complex both contribute to individual odors in congenic inbred strains of rats. Behav. Genet..

[16] Singh P.B., Brown R.E., Roser B. (1987). MHC anti genes in urine as olfactory recognition cues. Nature.

[17] Singh P.B., Herbert J., Roser B., Arnott L., Tucker D.K., Brown R.E. (1990). Rearing rats in a germ-free environment eliminates their odors of individuality. J. Chem. Ecol..

[18] Freeman-Gallant C.R., Meguerdichian M., Wheelwright N.T., Sollecito S.V. (2003). Social pairing and female mating fidelity predicted by restriction fragment length polymorphism similarity at the major histocompatibility complex in a songbird. Mol. Ecol..

[19] Richardson D.S., Komdeur J., Burke T., von Schantz T. (2005). MHC-based patterns of social and extra-pair mate choice in the Seychelles warbler. Proc. R. Soc. B.

[20] Bonneaud C., Chastel O., Federici P., Westerdahl H., Sorci G. (2006). Complex MHC-based mate choice in a wild passerine. Proc. R. Soc. B.

[21] Rekdal S.L., Anmarkrud J.A., Lifjeld J.T., Johnsen A. (2019). Extra-pair mating in a passerine bird with highly duplicated major histocompatibility complex class II: Preference for the golden mean. Mol. Ecol..

[22] Griggio M., Biard C., Penn D.J., Hoi H. (2011). Female house sparrows “count on” male genes: Experimental evidence for MHC-dependent mate preference. BMC Evol. Biol..

[23] Strandh M., Westerdahl H., Pontarp M., Canbäck B., Dubois M.-P., Miquel C., Taberlet P., Bonnadonna F. (2012). Major histocompatibility complex II compatibility, but not class I, predicts mate choice in a bird with highly developed olfaction. Proc. R. Soc. B.

[24] Leclaire S., Strandh M., Mardon J., Westerdahl H., Bonadonna F. (2017). Odour-based discrimination of similarity at the major histocompatibility complex in birds. Proc. R. Soc. B.

[25] Leclaire S. (2019). Odor-based mate choice in birds. Chem. Senses.

[26] Grieves L.A., Gloor G.B., Bernards M.A., MacDougall-Shackleton E.A. (2019). Songbirds show odour-based discrimination of similarity and diversity at the major histocompatibility complex. Anim. Behav..

[27] Baratti M., Dessi-Fulgheri F., Ambrosini R., Bonosoli-Alquati A., Caprioli M., Goti E., Matteo A., Monnanni R., Ragionieri L., Ristori E. (2012). MHC genotype predicts mate choice in the ring-necked pheasant Phasianus colchicus. J. Evol. Biol..

[28] Landry C., Garant D., Duchesne P., Bernatchez L. (2001). “Good genes as heterozygosity”: The major histocompatibility complex and mate choice in Atlantic salmon (*Salmo salar*). Proc. R. Soc. Lond. B.

[29] Reusch R.B.H., Häberli M.A., Aeschlimann P.B., Milinski M. (2001). Female sticklebacks count alleles in a strategy of sexual selection explaining MHC polymorphism. Nature.

[30] Milinski M., Griffiths S.W., Wegner K.M., Reusch T.B.H., Haas-Assenbaum A., Boehm T. (2005). Mate choice decisions of stickleback females predictably modified by MHC peptide ligands. Proc. Natl. Acad. Sci. USA.

[31] Milinski M., Griffiths S.W., Reusch T.B.H., Boehm T. (2010). Costly major histocompatibility complex signals produced only by reproductively active males, but not females, must be validated by a ‘maleness signal’ in threespined sticklebacks. Proc. R. Soc. B.

[32] Andreou D., Eizaguirre C., Boehm T., Milinski M. (2017). Mate choice in sticklebacks reveals that immunogenes can drive ecological speciation. Behav. Ecol..

[33] Gahr C.L., Boehm T., Milinski M. (2018). Female assortative mate choice functionally validates synthesized male odours of evolving stickleback river-lake ecotypes. Biol. Lett..

[34] Gahr C.L., Boehm T., Milinski M. (2021). Male validation factor for three-spined stickleback (*Gasterosteus aculeatus*) mate choice likely evolutionarily conserved since 50 thousand years. Ethol. Ecol. Evol..

[35] Eizaguirre C., Yeates S.E., Lenz T.L., Kalbe M., Milinski M. (2009). MHC-based mate choice combines good genes and maintenance of MHC polymorphism. Mol. Ecol..

[36] Eizaguirre C., Lenz T.L., Sommerfeld R.D., Harrod C., Kalbe M., Milinski M. (2011). Parasite diversity, patterns of MHC II variation and olfactory based mate choice in diverging three-spined stickleback ecotypes. Evol. Ecol..

[37] Forsberg L.A., Dannewitz J., Petersson E., Grahn M. (2007). Influence of genetic dissimilarity in the reproductive success and mate choice of brown trout–females fishing for optimal MHC dissimilarity. J. Evol. Biol..

[38] Olsson M., Madsen T., Nordby J., Wapstra E., Ujvari B., Wittsel H. (2003). Major histocompatibility complex and mate choice in sand lizards. Proc. R. Soc. Lond. B.

[39] Kloch A., Babik W., Bajer A., Sinski E., Radwan J. (2010). Effects of an MHC-DRB genotype and allele number on the load of gut parasites in the bank vole Myodes glareolus. Mol. Ecol..

[40] Schwensow N., Eberle M., Sommer S. (2008). Compatibility counts: MHC-associated mate choice in a wild promiscuous primate. Proc. R. Soc. B.

[41] Gilbert A.N., Yamazaki K., Beauchamp G.K., Thomas L. (1986). Olfactory discrimination of mouse strains (*Mus musculus*) and major histocompatibility types by humans (*Homo sapiens*). J. Comp. Psychol..

[42] Ober C., Weitkamp L.R., Cox N., Dytch H., Kostyu D., Elias S. (1997). HLA and mate choice in humans. Am. J. Hum. Genet..

[43] Wedekind C., Seebeck T., Bettens F., Paepke A.J. (1995). MHC-dependent mate preferences in humans. Proc. R. Soc. Lond. B.

[44] Wedekind C., Füri S. (1997). Body odour preferences in men and women: Do they aim for specific MHC combinations or simply heterozygosity?. Proc. R. Soc. Lond. B.

[45] Jacob S., McClintock M.K., Zelano B., Ober C. (2002). Paternally inherited HLA alleles are associated with women’s choice of male odor. Nature Genet..

[46] Roberts S.C., Gosling L.M., Carter V., Petrie M. (2008). MHC-correlated odour preferences in humans and the use of oral contraceptives. Proc. R. Soc. B.

[47] Yamazaki K., Beauchamp G.K., Bard G.K., Thomas L., Boyse E.A. (1982). Chemosensory recognition of phenotypes determined by the Tla and H-2k regions of chromosome 17 of the mouse. Proc. Natl. Acad. Sci. USA.

[48] Yamazaki K., Beauchamp G.K., Thomas L., Boyse E.A. (1984). Chemosensory identity of H-2 heterocygotes. J. Mol. Cell. Immunol..

[49] Yamazaki K., Beauchamp G.K., Imai Y., Bard J., Phelan S.P., Thomas L., Boyes E.A. (1990). Odortypes determined by the major histocompatibility complex in germfree mice. Proc. Natl. Acad. Sci. USA.

[50] Yamaguchi M., Yamazaki K., Beauchamp G.K., Bard J., Thomas L., Boyse E.A. (1981). Distinctive urinary odors gowerned by the major histocompatibility locus of the mouse. Proc. Natl. Acad. Sci. USA.

[51] Penn D., Potts W.K. (1998). Untrained mice discriminate MHC-determined odors. Physiol. Behav..

[52] Penn D.J., Potts W.K. (1998). How do major histocompatibility complex genes influence odor and mating preferences?. Adv. Immunol..

[53] Penn D.J., Potts W.K. (1998). Chemical signals and parasite-mediated sexual selection. Trends Ecol. Evol..

[54] Singer A.G., Beauchamp G.K., Yamazaki K. (1997). Volatile signals of the major histocompatibility complex in male mouse urine. Proc. Natl. Acad. Sci. USA.

[55] Yamazaki K., Beauchamp G., Singer A., Bard J., Boyse E. (1999). Odortypes: Their origin and composition. Proc. Natl. Acad. Sci. USA.

[56] Yamazaki K., Singer A., Beauchamp G. (1999). Origin, function and chemistry of H-2 regulated odorants. Genetica.

[57] Radwan J., Tkacz A., Kloch A. (2008). MHC and preferences for male odour in the bank vole. Ethology.

[58] Milinski M., Croy I., Hummel T., Boehm T. (2013). Major histocompatibility complex peptide ligands as olfactory cues in human body odour assessment. Proc. R. Soc. B..

[59] Klein J., Horejsi V. (1997). Immunology.

[60] Kubinak J.L., Stephens W.Z., Soto R., Petersen C., Chiaro T., Gogokhia L., Bell R., Ajami N.J., Petrosino J.F., Morrison L. (2015). MHC variation sculpts individualized microbial communities that control susceptibility to enteric infection. Nature Comms..

[61] Bolnick D.I., Snowberg L.K., Caporaso J.G., Lauber C., Knight R., Stutz W.E. (2014). Major Histocompatibility Complex class IIb polymorphism influences gut microbiota composition and diversity. Mol. Ecol..

[62] Grieves L.A., Gloor G.B., Bernards M.A., MacDougall-Shackleton E.A. (2021). Preen gland microbiota covary with major histocompatibility complex genotype in a songbird. R. Soc. Open Sci..

[63] Singh P.B. (1999). The present state of the ‘carrier hypothesis’ for chemosensory recognition of genetic individuality. Genetica.

[64] Douglas A.E., Dobson A.J. (2013). New synthesis: Animal communication mediated by microbes: Fact or fantasy?. J. Chem. Ecol..

[65] Falk K., Rötzschke O., Stevanovic S., Jung G., Rammensee H.G. (1991). Allele-specific motifs revealed by sequencing of self-peptides eluted from MHC molecules. Nature.

[66] Koch M., Camp S., Collen T., Avila D., Salomonsen J., Wallny H.J., Van Heteren A., Hunt L., Jacob J.P., Johnston F. (2007). Structures of an MHC class I molecule from B21 chickens illustrate promiscuous peptide binding. Immunity.

[67] Chappell P., Meziane E.K., Harrison M., Magiera L., Hermann C., Mears L., Wrobel A.G., Durant C., Nielsen L.L., Buus S. (2015). Expression levels of MHC class I molecules are inversely correlated with promiscuity of peptide binding. elife.

[68] Janeway C., Travers P., Walport M., Shlomchic M. (2001). Immunobiology: The Immune System in Heath and Disease.

[69] Singh P.B., Brown R.E., Roser B.J. (1988). Class I transplantation antigens in solution in the body fluids and the urine: Individuality signals to the environment. J. Exp. Med..

[70] Singh P.B. (2001). Chemosensation and genetic individuality. Reproduction.

[71] Savage D.C. (1977). Microbial ecology of the gastrointestinal tract. Annu. Rev. Microbiol..

[72] Yamazaki K., Beauchamp G.K., Thomas L., Boyse E.A. (1985). Thehematopoietic system is a source of odorants that distinguish majorhistocompatibility types. J. Exp. Med..

[73] Katz D.H., Skidmore B.J., Katz L.R., Bogowitz C.A. (1978). Adaptive differentiation of murine lymphocytes. I. Both T and B lymphocytes differentiating in F1 transplanted to parental chimeras manifest preferential cooperative activity for partner lymphocytes derived from the sameparental type corresponding to the chimeric host. J. Exp. Med..

[74] Kwak J., Willse A., Preti G., Yamazaki K., Beauchamp G.K. (2010). In search of the chemical basis for MHC odortypes. Proc. R. Soc. B.

[75] Parham P., Ohta T. (1996). Population biology of antigen presentation by MHC class I molecules. Science.

[76] Lawlor D.A., Zemmour J., Ennis P.D., Parham P. (1990). Evolution of class-I MHC genes and proteins: From natural-selection to thymic selection. Annu. Rev. Immunol..

[77] Nowak M.A., Tarczyhornoch K., Austyn J.M. (1992). The optimal number of major histocompatibility complex-molecules in an individual. Proc. Natl. Acad. Sci. USA.

[78] De Boer R.J., Perelson A.S. (1993). How diverse should the immune system be?. Proc. R. Soc. Lond. B.

[79] Woelfing B., Traulsen A., Milinski M., Boehm T. (2009). Does intra-individual major histocompatibility complex diversity keep a golden mean?. Phil. Trans. R. Soc. B.

[80] Feulner P.G.D., Chain F.J.J., Panchal M., Huang Y., Eizaguirre C., Kalbe M., Lenz T.L., Samonte I.E., Stoll M., Bornberg-Bauer E. (2015). Genomics of divergence along a continuum of parapatric population differentiation. PLoS Genet..

[81] Hamilton W.D., Zuk M. (1982). Heritable true fitness and bright birds: A role for parasites?. Science.

[82] Milinski M., Bakker T.C.M. (1990). Female sticklebacks use male coloration in mate choice and hence avoid parasitized males. Nature.

[83] O’Connor E.A., Strandh M., Hasselquist D., Nilsson J.A., Westerdahl H. (2016). The evolution of highly variable immunity genes across a passerine bird radiation. Mol. Ecol..

[84] Biedrzycka A., O’Connor E., Sebastian A., Migalska M., Radwan J., Zajac T., Bielanski W., Solarz W., Cmiel A., Westerdahl H. (2017). Extreme MHC class I diversity in the sedge warbler (*Acrocwphalus schoeno* baenus); selection patterns and allelic divergence suggest that different genes have different functions. BMC Evol. Biol..

[85] Wegner K.M., Reusch T.B.H., Kalbe M. (2003). Multiple parasites are driving major histocompatibility complex polymorphism in the wild. J. Evol. Biol..

[86] Wegner K.M., Kalbe M., Kurtz J., Reusch T.B.H., Milinski M. (2003). Parasite selection for immunogenetic optimality. Science.

[87] Kalbe M., Eizaguirre C., Dankert I., Reusch T.B.H., Sommerfeld R.D., Wegner K.M., Milinski M. (2009). Lifetime reproductive success is maximized with optimal major histocompatibility complex diversity. Proc. R. Soc. B.

[88] Wegner K.M., Kalbe M., Milinski M., Reusch T.B.H. (2008). Mortality selection during the 2003 European heat wave in three-spined sticklebacks: Effects of parasites and MHC genotype. BMC Evol. Biol..

[89] Bonneaud C., Mazuc J., Chastel O., Westerdahl H., Sorci G. (2004). Terminal investment induced by immune challenge and fitness traits associated with major histocompatibility complex in the house sparrow. Evolution.

[90] Aeschlimann P.B., Häberli M.A., Reusch T.B.H., Boehm T., Milinski M. (2003). Female sticklebacks Gasterosteus aculeatus use self-reference to optimize MHC allele number during mate selection. Behav. Ecol. Sociobiol..

[91] Boehm T. (2006). Co-evolution of a primordial peptide-presentation system and cellular immunity. Nature Rev. Immunol..

[92] Boehm T., Zufall F. (2006). MHC peptides and the sensory evaluation of genotype. Trends Neurosci..

[93] Boehm T. (2013). A whiff of genome. Nature.

[94] Leinders-Zufall T., Brennan P., Widmayer P., Chandramani P.S., Maul-Pavicic A., Jäger M., Xiao-Hong L., Breer H., Zufall F., Boehm (2004). T. MHC class I peptides as chemosensory signals in the vomeronasal organ. Science.

[95] Sturm T., Leinders-Zufall T., Macek B., Walzer M., Jung S., Poemmerl B., Stevanovic S., Zufall F., Overath P., Rammensee H.-G. (2013). Mouse urinary peptides provide a molecular basis for genotype discrimination by nasal sensory neurons. Nat. Commun..

[96] Spehr M., Kelliher K.R., Li X.-H., Boehm T., Leinders-Zufall T., Zufall F. (2006). Essential role of the main olfactory system in social recognition of major histocompatibility complex peptide ligands. J. Neurosci..

[97] Bruce H.M. (1959). An exteroceptive block to pregnancy in the mouse. Nature.

[98] Yamazaki K., Beauchamp G.K., Matzuzaki O., Kupniewski D., Bard J., Thomas L., Boyse E.A. (1986). Influence of a genetic difference confined to mutation of H-2K on the incidence of pregnancy block in mice. Proc. Natl. Acad. Sci. USA.

[99] Milinski M. (2003). The function of mate choice in sticklebacks optimizing MHC genetics. J. Fish. Biol..

[100] Milinski M., Wedekind C. (2001). Evidence for MHC-correlated perfume preferences in humans. Behav. Ecol..

[101] Schubert N., Winternitz J.C., Nichols H.L. (2021). How can the MHC mediate social odor via the microbiota community?. Behav. Ecol..

[102] Winternitz J.C., Schubert N., Nichols H. (2021). Let’s keep alternative hypotheses on the table: A response to comments on Schubert et al. Behav. Ecol..

[103] Milinski M. (2021). MHC mediates social odor via microbiota—It cannot work: A comment on Schubert et al.. Behav. Ecol..

[104] Wedekind C. (1994). Mate choice and maternal selection for specific parasite resistances before, during and after fertilization. Phil. Trans. R. Soc. B Biol. Sci..

[105] Jordan W.C., Bruford M.W. (1998). New perspective on mate choice and the MHC. Heredity.

[106] Tregenza T., Wedell N. (2000). Genetic compatibility, mate choice and patterns of parentage: Invited review. Mol. Ecol..

[107] Birkhead T.R., Pizzari T. (2002). Postcopulatory sexual selection. Nat. Rev. Genet..

[108] Ziegler A., Kentenich H., Uchanska-Ziegler B. (2005). Female choice and the MHC. Trends Immunol..

[109] Hutter H., Dohr G. (1998). HLA expression on immature and mature human germ cells. J. Reprod. Immunol..

[110] Fernandes N., Cooper J., Sprinks M., AbdElrahman M., Fiszer D., Kurpisz M., Dealtry G. (1999). A critical review of the role of the major histocompatibility complex in fertilization, preimplantation development and feto-maternal interactions. Hum. Reprod. Update.

[111] Mori T., Sato E., Baba T., Seiichi T., Mori E. (2000). Molecular and immunological approaches to mammalian fertilization. J. Reprod. Immunol..

[112] Mori T., Guo M.W., Mori E., Shindo Y., Mori N., Fukuda A., Mori T. (1990). Expression of class II major histocompatibility complex antigen on mouse sperm and its roles in fertilization. Am. J. Reprod. Immunol..

[113] Yanagimachi R., Cherr G., Matsubara T., Andoh T., Harumi T., Vines C., Pillai M., Griffin F., Matsubara H., Weatherby T. (2013). Sperm attractant in the micropyle region of fish and insect eggs. Biol. Reprod..

[114] Yanagimachi R., Harumi T., Matsubara H., Yan W., Yuan S., Hirohashi N., Iida T., Yamaha E., Arai K., Matsubara T. (2017). Chemical and physical guidance of fish spermatozoa into the egg through the micropyle. Biol. Reprod..

[115] Yeates S.E., Einum S., Fleming I.A., Megens H.J., Stet R.J., Hindar K., Holt W.V., Van Look K.J., Gage M.J. (2009). Atlantic salmon eggs favour sperm in competition that have similar major histocompatibility alleles. Proc. R. Soc. B Biol. Sci..

[116] Geßner C., Nakagawa S., Zavodna M., Gemmell N.J. (2017). Sexual selection for genetic compatibility: The role of the major histocompatibility complex on cryptic female choice in Chinook salmon (*Oncorhynchus tshawytscha*). Heredity.

[117] Gasparini C., Congiu L., Pilastro A. (2015). MHC similarity and sexual selection: Different doesn’t always mean attractive. Mol. Ecol..

[118] Løvlie H., Gillingham M.A.F., Worley K., Pizzari T., Richardson D.S. (2013). Cryptic female choice favours sperm from major histocompatibility complex-dissimilar males. Proc. R. Soc. B Biol. Sci..

[119] Wedekind C., Chapuisat M., Macas E., Rülike T. (1996). Non-random fertilization in mice correlates with the MHC and something else. Heredity.

[120] Rülike T., Chapuisat M., Homberger F.R., Macas E., Wedekind C. (1998). MHC-genotype of progeny influenced by parental infection. Proc. R. Soc. B Biol. Sci..

[121] Firman R.C., Simmons L.W. (2015). Gametic interactions promote inbreeding avoidance in house mice. Ecol. Lett..

[122] Lenz T.L., Hafer N., Samonte I.E., Yeates S.E., Milinski M. (2018). Cryptic haplotype-specific gamete selection yields offspring with optimal MHC immune genes. Evolution.

